# Attenuation of Rheumatoid Inflammation by Sodium Butyrate Through Reciprocal Targeting of HDAC2 in Osteoclasts and HDAC8 in T Cells

**DOI:** 10.3389/fimmu.2018.01525

**Published:** 2018-07-06

**Authors:** Da Som Kim, Jeong-Eun Kwon, Seung Hoon Lee, Eun Kyung Kim, Jun-Geol Ryu, Kyung-Ah Jung, Jeong-Won Choi, Min-Jung Park, Young-Mee Moon, Sung-Hwan Park, Mi-La Cho, Seung-Ki Kwok

**Affiliations:** ^1^The Rheumatism Research Center, College of Medicine, Catholic Research Institute of Medical Science, The Catholic University of Korea, Seoul, South Korea; ^2^Division of Immunology, Department of Microbiology and Immunobiology, Harvard Medical School, Boston, MA, United States; ^3^Impact Biotech, Seoul, South Korea; ^4^Division of Rheumatology, Department of Internal Medicine, Seoul St. Mary’s Hospital, College of Medicine, The Catholic University of Korea, Seoul, South Korea; ^5^Laboratory of Immune Network, Conversant Research Consortium in Immunologic Disease, College of Medicine, The Catholic University of Korea, Seoul, South Korea; ^6^College of Medicine, The Institute for Aging and Metabolic Diseases, The Catholic University of Korea, Seoul, South Korea

**Keywords:** sodium butyrate, rheumatoid arthritis, histone deacetylases, glucocorticoid receptor, ERRα

## Abstract

Rheumatoid arthritis (RA) is a systemic autoimmune disease caused by both genetic and environmental factors. Recently, investigators have focused on the gut microbiota, which is thought to be an environmental factor that affects the development of RA. Metabolites secreted by the gut microbiota maintain homeostasis in the gut through various mechanisms [e.g., butyrate, which is one of the major metabolites of gut microbiota, exerts an anti-inflammatory effect by activating G-protein-coupled receptors and inhibiting histone deacetylases (HDACs)]. Here, we focused on the inhibition of the HDACs by butyrate in RA. To this end, we evaluated the therapeutic effects of butyrate in an animal model of autoimmune arthritis. The arthritis score and incidence were lower in the butyrate-treated group compared to the control group. Also, butyrate inhibited HDAC2 in osteoclasts and HDAC8 in T cells, leading to the acetylation of glucocorticoid receptors and estrogen-related receptors α, respectively. Additionally, control of the T_H_17/T_reg_ cell balance and inhibition of osteoclastogenesis were confirmed by the changes in target gene expression. Interleukin-10 (IL-10) produced by butyrate-induced expanded T_reg_ cells was critical, as treatment with butyrate did not affect inflammatory arthritis in IL-10-knockout mice. This immune-cell regulation of butyrate was also detected in humans. These findings suggest that butyrate is a candidate agent for the treatment of RA.

## Introduction

Rheumatoid arthritis (RA) is an autoimmune disease of unknown etiology that involves joint destruction (1). An intestinal imbalance is involved in RA development, and gut bacteria have various effects on RA (2, 3). Because the gut microbiota plays an important role in maintaining homeostasis, any imbalance can lead to the development of various diseases and systemic effects. In addition to protecting the intestinal surface from pathogens, it also participates in digestion and affects bone density and immune system development (4). Indeed, the gut microbiota is also involved in bone metabolism (5) and secreted metabolites modulate the T_H_17/T_reg_ cell balance (6). For example, segmented filamentous bacteria secrete serum amyloid A, which induces differentiation of T_H_17 cells (7, 8). Several bacterial species secrete short-chain fatty acids (SCFAs) (9, 10) (e.g., acetate, butyrate, and propionate), which induce differentiation of T_reg_ cells from naïve CD4^+^ T cells (9, 11). SCFAs have immunomodulatory activity. For instance, butyrate exerts an anti-inflammatory effect by downregulating IL-12 and upregulating IL-10 (12). Therefore, butyrate may have a therapeutic effect in immune-mediated inflammatory diseases. Indeed, butyrate reportedly has therapeutic efficacy in various animal models of inflammatory diseases [e.g., dextran sodium sulfate-induced colitis, obesity, and graft-versus-host disease (GvHD)] (13–16). Regarding GvHD, allogeneic bone marrow transplant-recipient mice (C57BL/6J → BALB/c) exhibited significantly reduced butyrate levels in intestinal tissue. Butyrate treatment strengthened the intestinal epithelial barrier, indicating that butyrate has immunomodulatory activity. Butyrate also inhibits histone deacetylase (HDAC). In inflammatory arthritis, although the abundance of *Faecalibacterium prausnitzii*, a butyrate-producing bacterium, is reduced in enthesitis-related arthritis (17), the direct therapeutic effect of butyrate on RA is unclear. Therefore, both the therapeutic effect of butyrate in RA and the mechanism underlying this effect should be investigated. Here, we assessed the therapeutic effect of butyrate in an animal model of RA. To identify the mechanism underlying its anti-arthritic effect, we also examined the effect of butyrate on effector T-cell differentiation and osteoclastogenesis. Finally, we verified the mechanism of the effect of butyrate.

## Materials and Methods

### Animals

DBA/1J mice, C57BL/6 mice, and IL-10-knockout (KO) (Orient) mice were maintained in groups of five in polycarbonate cages in a specific-pathogen-free environment. They were provided with access to standard mouse chow (Ralston Purina, Gray Summit, MO, USA) and water *ad libitum*. All experimental procedures were approved by the Animal Research Ethics Committee at the Catholic University of Korea (approval numbers, 2017-0070-02 and 2018-0014-01).

### Induction of Collagen-Induced Arthritis (CIA) and Treatment With Butyrate

Collagen-induced arthritis was generated in male DBA/1J mice or IL-10-KO mice. Mice were immunized with 100 µg of chicken type II collagen (CII) (Chondrex Inc., Redmond, WA, USA) dissolved overnight in 0.1 N acetic acid (4 mg/mL) in complete Freund’s adjuvant (CFA). The immunizations were performed intradermally into the base of the tail. Two weeks after primary immunization, mice were boosted with 100 µg of CII in incomplete Freund’s adjuvant (Chondrex Inc.). CIA mice were treated with 100 mg/kg sodium butyrate in saline or with saline alone *via* intraperitoneal injections three times per week beginning on day 17 after primary immunization. Butyrate was administered during the entire study period.

### Clinical Scoring of Arthritis

Mice were considered to have arthritis when significant changes in redness and/or swelling were noted in the digits or in other parts of the paws. Knee-joint inflammation was scored visually after dissection on a scale from 0 to 4 (0, uninflamed; 1, minimal; 2, mild; 3, moderate; and 4, severe inflammation). Scoring was performed by two independent observers.

### Histological Analysis

Histological analysis was performed to determine the extent of joint damage. Mice joint tissues were fixed in 4% paraformaldehyde, decalcified in 10% ethylenediaminetetraacetic acid solution, embedded in paraffin, and sectioned. The sections were deparaffinized using xylene and dehydrated through an alcohol gradient. Endogenous peroxidase activity was quenched with methanol–3% H_2_O_2_. Sections were staining using hematoxylin and eosin (H&E), safranin O, or tartrate-resistant acid phosphatase (TRAP).

### Confocal Microscopy

Naïve CD4^+^ T cells were placed in the appropriate well of a cytospin chamber (Thermo Fisher Scientific, MI, USA) and centrifuged at 700 × *g* for 3 min. Tissue cryosections (7-µm thick) or naïve CD4^+^ T cells cultured under T_H_17 differentiation conditions were fixed with methanol–acetone and stained with fluorescein isothiocyanate (FITC)-, PE-, PerCP-Cy5.5-, or allophycocyanin (APC)-conjugated monoclonal antibodies against mouse CD4, CD25, IL-17, Foxp3, IL-10, DAPI, CPTIA, and NR1D1 (eBioscience, San Diego, CA, USA). After an overnight incubation at 4°C, the stained sections were visualized by confocal microscopy (LSM 510 Meta; Zeiss).

### Immunohistochemistry

Immunohistochemistry was performed using the Vectastain ABC kit (Vector Laboratories, Burlingame, CA, USA). Tissue sections were incubated overnight at 4°C with primary antibodies against IL-1β, IL-6, IL-17, TNF-α, CD68, and secretory leukocyte protease inhibitor (SLPI); probed with a biotinylated secondary antibody; and stained with a streptavidin-peroxidase complex for 1 h. DAB chromogen (Dako, Carpinteria, CA, USA) was added as a substrate, and the samples were visualized by microscopy (Olympus, Center Valley, PA, USA).

### Quantification of CII-Specific Antibodies

Blood was obtained from the orbital sinus of CIA mice, and the serum was stored at −20°C until used. The serum levels of antibodies to CII-specific mouse IgG, IgG1, IgG2a, and IgG3 were measured using enzyme-linked immunosorbent assay kits (Bethyl Laboratories, Montgomery, TX, USA).

### Mouse *In Vitro* Osteoclastogenesis

Bone marrow-derived monocyte/macrophages (BMMs) were isolated from the tibias and femurs of CIA mice by flushing the bone-marrow cavity with minimum essential medium-α (Invitrogen, Carlsbad, CA, USA). The cells were incubated for 6 h to separate nonadherent and adherent cells. Non-adherent cells were seeded in 48-well plates at 2 × 10^5^ cells/well and cultured in the presence of 10 ng/mL rh M-CSF (R&D Systems, Minneapolis, MN, USA) for 3 days to form macrophage-like osteoclast precursor cells (preosteoclasts). Three days later, the nonadherent cells were washed out, and preosteoclasts were cultured in the presence of 10 ng/mL M-CSF, 50 ng/mL RANKL (Peprotech, London, UK), and various concentrations of sodium butyrate for 4 days to generate osteoclasts. On day 2, the medium was replaced with fresh medium containing M-CSF, RANKL, and sodium butyrate.

For osteoclast staining for SLPI, BMM of CIA-induced mice were separated into non-adherent and adherent cells, and the former were cultured with M-CSF on 8-well cell culture slides for 3 days. After 3 days, preosteoclasts were removed and cultured in the presence of M-CSF and RANKL. After 1 day, medium was washed and incubated for 1 day in the presence of 100 µM and 1 mM sodium butyrate.

### Mouse *Ex Vivo* Osteoclastogenesis

Bone marrow-derived monocyte/macrophages were isolated from the tibias and femurs of IL-10-KO mice 11 weeks after CIA induction. These cells were seeded under the same conditions used for mouse *in vitro* osteoclastogenesis; after 3 days, they were stimulated with the same concentrations of M-CSF and RANKL. Preosteoclasts were cultured for 3 days to generate osteoclasts; on day 2, the medium was replaced with fresh medium containing M-CSF and RANKL.

### Human *In Vitro* Osteoclastogenesis

Peripheral blood mononuclear cells (PBMCs) obtained from normal healthy humans were separated from buffy coats using Ficoll-Hypaque (Pharmacia Biotech, Piscataway, NJ, USA). Red blood cells were removed, and the cells were seeded into 24-well plates at 5 × 10^5^ cells/well and incubated at 37°C for 2 h to separate non-adherent and adherent cells. The adherent cells were washed with phosphate-buffered saline and cultured with 100 ng/mL M-CSF for 3 days. After 3 days, these preosteoclasts were cultured in the presence of 25 ng/mL M-CSF, 30 ng/mL RANKL, and various concentrations of sodium butyrate for 6 days to generate osteoclasts. On day 3, the medium was replaced with fresh medium containing M-CSF, RANKL, and sodium butyrate. All human experimental procedures were approved by the Ethics Committee of Seoul St. Mary’s Hospital (Seoul, Republic of Korea, KC17TNSI0570) and written informed consent was obtained.

### TRAP Staining

A commercial TRAP staining kit (Sigma-Aldrich, St. Louis, MO, USA) was used according to the manufacturer’s instructions. TRAP-positive multinucleated cells (MNCs) containing at least three nuclei were counted as osteoclasts.

### Real-Time Polymerase Chain Reaction (PCR)

Messenger RNA (mRNA) was extracted using TRI Reagent (Molecular Research Center, Inc., Cincinnati, OH, USA) according to the manufacturer’s instructions. Complementary DNA was synthesized using a Super Script Reverse Transcription system (TaKaRa, Shiga, Japan). A Light-Cycler 2.0 instrument (software version 4.0; Roche Diagnostics) was used for PCR amplification. All reactions were performed using the LightCycler FastStart DNA Master SYBR Green I mix (TaKaRa) following the manufacturer’s instructions. The following primers were used: Carbonic anhydrase II, 5′-TGG-TTC-ACT-GGA-ACA-CCA-AA-3′ (sense) and 5′-AGC-AAG-GGT-CGA-AGT-TAG-CA-3′ (antisense); TRAP, 5′-TCC-TGG-CTC-AAA-AAG-CAG-TT-3′ (sense) and 5′-ACA-TAG-CCC-ACA-CCG-TTC-TC-3′ (antisense); integrin β3, 5′-CCA-CAC-GAG-GCG-TGA-ACT-3′ (sense) and 5′-CTT-CAG-GTT-ACA-TCG-GGG-TGA-3′ (antisense); DC-stamp, 5′-GCA-CGC-AAT-CGC-GTC-AAC-T-3′ (sense) and 5′-AGG-CAT-TCC-GTC-TGC-TTT-GA-3′ (antisense); matrix metalloproteinase-9 (MMP-9), 5′-CTG-TCC-AGA-CCA-AGG-GTA-CAG-CCT-3′ (sense) and 5′-GAG-GTA-TAG-TGG-GAC-ACA-TAG-TGG-3′ (antisense); calcitonin receptor, 5′-CGG-ACT-TTG-ACA-CAG-CAG-AA-3′ (sense) and 5′-AGC-AGC-AAT-CGA-CAA-GGA-GT-3′ (antisense); HDAC1, 5′-CTA-TGC-TGT-GAA-CTA-CCC-ACT-G-3′ (sense) and 5′-GGA-ATC-TGA-GCC-ACA-CTG-TAA-G-3′ (antisense); HDAC2, 5′-CAT-GGC-GTA-CAG-TCA-AGG-AG-3′ (sense) and 5′-AGC-AAG-TTA-TGA-GTC-ATC-CGG-3′ (antisense); HDAC3, 5′-TGT-CTC-AAT-GTG-CCC-TTA-CG-3′ (sense) and 5′-CCT-AAT-CGA-TCA-CAG-CCC-AG-3′ (antisense); HDAC4, 5′-AGC-ACT-GAG-AAT-GGC-ATC-G-3′ (sense) and 5′-TGA-TGT-TGG-GTA-AGG-ATG-GTG-3′ (antisense); HDAC5, 5′-TTC-AAC-TCC-GTA-GCC-ATC-AC-3′ (sense) and 5′-GGA-TCG-TTG-TAG-AAT-GCT-TGC-3′ (antisense); HDAC6, 5′-TGC-CCA-CCT-AAC-CCA-TTT-G-3′ (sense) and 5′-AAG-CAC-TGA-TTC-CCT-TAG-CC-3′ (antisense); HDAC7, 5′-TCA-AAC-TGG-ATA-ACG-GGA-AGC-3′ (sense) and 5′-CCA-GAT-GGT-GTC-AGT-ATC-GAC-3′ (antisense); HDAC8, 5′-ACC-GAA-TCC-AGC-AAA-TCC-TC-3′ (sense) and 5′-CAG-TCA-CAA-ATT-CCA-CAA-ACC-G-3′ (antisense); HDAC9, 5′-AGG-ATG-ATG-ATG-CCT-GTG-GTG-GAT-3′ (sense) and 5′-GAG-TTG-TGC-TTG-ATG-CTG-CCT-TGT-3′ (antisense); HDAC10, 5′-CTG-TCA-ATT-TGC-CCT-GGA-AC-3′ (sense) and 5′-CCC-CGA-TAG-CAG-AGT-CAA-ATC-3′ (antisense); HDAC11, 5′-GCT-GGG-AAA-TGG-GGC-AAG-GTG-A-3′ (sense) and 5′-AGC-TCG-TTG-AGA-TAG-CGC-CTC-GT-3′ (antisense); TNF-α, 5′-AAG-CCT-GTA-GCC-CAC-GTC-GTA-3′ (sense) and 5′-GGC-ACC-ACT-AGT-TGG-TTG-TCT-TTG-3′ (antisense); Foxp3, 5′-GGC-CCT-TCT-CCA-GGA-CAG-A-3′ (sense) and 5′-GCT-GAT-CAT-GGC-TGG-GTT-GT-3′ (antisense); CTLA4, 5′-TGA-CCC-AAC-CTT-CAG-TGG-TG-3′ (sense) and 5′-TTT-GGT-CAT-TTG-TCT-GCC-GC-3′ (antisense); CPTIA, 5′-GAT-CTA-CAA-TTC-CCC-TCT-GCT-C-3′ (sense) and 5′-AGC-CAG-ACC-TTG-AAG-TAA-CG-3′ (antisense); NR1D1, 5′-GCC-ATG-TTT-GAC-TTC-AGC-G-3′ (sense) and 5′-AAT-TCT-CCA-TTC-CCG-AGC-G-3′ (antisense); SLPI, 5′-GGC-CTT-TTA-CCT-TTC-ACG-GTG-3′ (sense) and 5′-GGC-TCC-GAT-TTT-GAT-AGC-ATC-AT-3′ (antisense); IL-10, 5′-GGC-CCA-GAA-ATC-AAG-GAG-CA-3′ (sense) and 5′-AGA-AAT-CGA-TGA-CAG-CGC-CT-3′ (antisense); and β-actin, 5′-GAA-ATC-GTG-CGT-GAC-ATC-AAA-G-3′ (sense), and 5′-TGT-AGT-TTC-ATG-GAT-GCC-ACA-G-3′ (antisense). All mRNA levels were normalized to that of β-actin.

### Murine T-Cell Isolation and Differentiation

To purify splenic CD4^+^ T cells, splenocytes were incubated with CD4-coated magnetic beads and isolated using magnetic-activated cell sorting (MACS) separation columns (Miltenyi Biotec, Bergisch Gladbach, Germany). MACS-sorted CD4^+^ T cells were sorted to obtain naïve CD4^+^ T cells by selecting for CD4^+^CD62L^high^CD44^low^. To establish T_H_17 cell-polarizing conditions, the cells were stimulated with anti-CD3 (0.5 µg/mL), anti-CD28 (1 µg/mL), anti-interferon-γ (anti-IFN-γ) (1 µg/mL), anti-IL-4 (2 µg/mL), IL-6 (40 ng/mL), and transforming growth factor-β (TGF-β) (2 ng/mL) for 3 days. Recombinant mouse IL-6 and antibodies to IFN-γ and IL-4 were purchased from R&D Systems, and TGF-β was purchased from PeproTech.

### Human CD4^+^ T-Cell Isolation and Differentiation

CD4^+^ T cells were isolated from human PBMCs using a CD4^+^ T-cell isolation kit (Miltenyi Biotec) according to the manufacturer’s instructions. Th0 cells were stimulated with anti-CD3 (0.5 µg/mL) and anti-CD28 (1 µg/mL) with no added cytokines. To establish T_H_17 cell-polarizing conditions, the CD4^+^ T cells were stimulated with anti-CD3, anti-CD28, anti-IFN-γ (2 µg/mL), anti-IL-4 (2 µg/mL), IL-1β (20 ng/mL), and IL-6 (20 ng/mL) for 3 days. Recombinant human IL-1β and IL-6 and antibodies to IFN-γ and IL-4 were purchased from R&D Systems, and TGF-β was purchased from Peprotech.

### Flow Cytometry

For intracellular staining, cells were restimulated with 25 ng/mL phorbol 12-myristate 13-acetate and 250 ng/mL ionomycin (both from Sigma-Aldrich) for 4 h in the presence of GolgiStop (BD Biosciences, Sparks, MD, USA). Murine splenocytes were stained with surface PerCP-conjugated anti-CD4 (eBioscience) and APC-conjugated anti-CD25 (BioLegend, San Diego, CA, USA) antibodies. After fixation and permeabilization, cells were stained with FITC-conjugated anti-IL-17, APC-conjugated anti-IFN-γ, phycoerythrin (PE)-conjugated anti-IL-4, or PE-conjugated anti-Foxp3 antibodies (eBioscience). Human CD4^+^ T cells were stained with surface PE-Cy7-conjugated anti-CD4 and APC-conjugated anti-CD25 antibodies (BioLegend). After fixation and permeabilization, cells were stained with PE-conjugated anti-IL-17 or PE-conjugated anti-Foxp3 antibodies (eBioscience). Events were collected and analyzed with FlowJo software (Tree Star, Ashland, OR, USA).

### Western Blotting

The protein levels of p-STAT3 Y705, p-STAT3 S727, STAT3 (Cell Signaling, Danvers, MA, USA), and GAPDH (Abcam, Cambridge, MA, USA) were measured using a western blot system (SNAP i.d. Protein Detection System, Merck Millipore, Danvers, MD, USA). Total splenocytes or CD4^+^ T cells were preincubated in the presence or absence of sodium butyrate (100, 200, and 500 µM) for 2 h, cultured with IL-6 (10 ng/mL) for 1 h, and cell lysates were prepared. Protein concentrations were determined using the Bradford method (Bio-Rad, Hercules, CA, USA), and samples were separated on a sodium dodecyl sulfate polyacrylamide gel and transferred to a nitrocellulose membrane (Amersham Pharmacia, Uppsala, Sweden). The primary antibodies to p-STAT3 Y705, p–STAT3 S727, STAT3, and GAPDH were diluted in 0.1% skim milk in Tris-buffered saline and incubated for 20 min at room temperature. The membrane was washed and incubated with a horseradish peroxidase-conjugated secondary antibody for 20 min at room temperature.

### Immunoprecipitation

RAW 264.7 cells were seeded into 100-mm dishes at 1 × 10^6^ cells/dish and cultured overnight with M-CSF (10 ng/mL), RANKL (50 ng/mL), and sodium butyrate (500 µM). EL4 cells were seeded into 100-mm dishes at 1 × 10^6^ cells and cultured for 2 days in the presence of anti-CD3 (0.5 µg/mL), IL-6 (10 ng/mL), and sodium butyrate (500 µM). Cells were harvested in lysis buffer (150 mM NaCl, 20 mM Tris, 0.1% NP-40, 5 mM MgCl_2_, 10% glycerol, 300 nM TSA, 10 mM nicotinamide, 1× complete protease inhibitors; pH 8). Immunoprecipitation was performed using antibodies against glucocorticoid receptor (GR) and ERRα (Abcam) with rotation at 4°C for 1 h with Dynabeads^®^ Protein G (Thermo Fisher Scientific). Cell lysate was added, the system was rotated at 4°C for 2 h, and elution was performed. Immunoblotting was performed using anti-GR, -ERRα, and -acetyl lysine antibodies (Abcam).

### Statistical Analysis

Experiments were independently replicated at least twice, and representative and/or summary data are shown. Variation in the distribution of results was examined using the Shapiro–Wilk test. Data are presented as means ± SD. Comparisons of the numerical data obtained from two groups were performed by Student’s *t*-test or the Mann–Whitney *U*-test. Differences in the mean values of various groups were subjected to analysis of variance and a *post hoc* test. *p*-Values less than 0.05 (two-tailed) were considered indicative of statistical significance. All statistical analyses were performed using SAS software (version 9.2; SAS Institute, Cary, NC, USA).

## Results

### Butyrate Reduced the Severity of Arthritis in the CIA Mouse Model

**Figure 1:**
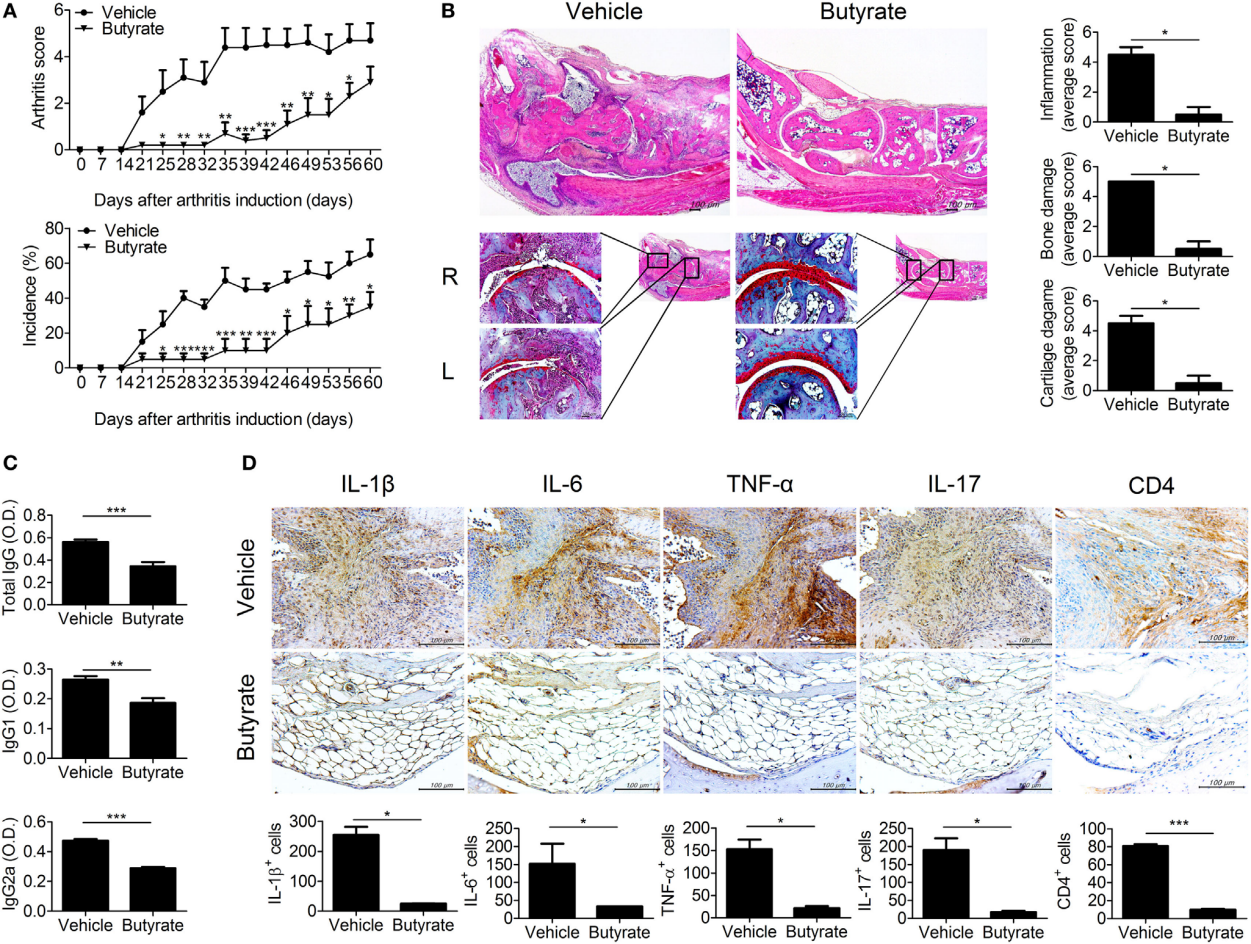
Butyrate mitigates disease exacerbations in collagen-induced arthritis (CIA) mice. **(A)** CII-induced DBA/1J mice were injected intraperitoneally with 100 mg/kg butyrate three times per week after 3 days of secondary immunization. The clinical score (upper panel) and incidence (lower panel) of arthritis in treated mice are presented (*n* = 5). **(B)** Histological staining of joint tissue sections with hematoxylin and eosin (original magnification, 40×) and safranin O (original magnification, 200×). Histological inflammation scores are shown (*n* = 3). **(C)** Seven weeks after generation of CIA, the serum concentrations of CII-specific IgG, IgG1, and IgG2a were evaluated. **(D)** Representative histological features of the joints of vehicle- and butyrate-treated mice (*n* = 3). Immunohistochemical staining for IL-1β, IL-6, TNF-α, IL-17, and CD4 are shown (original magnification, 400×). Graphs show the numbers of cells expressing IL-1β, IL-6, TNF-α, IL-17, and CD4. *p < 0.05, **p < 0.01, ***p < 0.001.

We administered butyrate to CIA mice. The arthritis scores and the incidence of arthritis were dramatically reduced by butyrate compared to vehicle treatment (Figure 1A). Histological evaluation of joint tissues showed that bone and cartilage damage was almost completely reversed by treatment with butyrate. The inflammation, bone damage, and cartilage damage scores were markedly reduced in the joints of butyrate-treated mice compared to vehicle-treated mice (Figure 1B). Serum CII-specific IgG, IgG1, and IgG2a levels were also reduced by administration of butyrate (Figure 1C). These data suggest that butyrate ameliorates CIA. Moreover, the expression levels of IL-17, IL-1β, IL-6, and TNF-α in joint tissues were markedly reduced by treatment with butyrate compared to vehicle (Figure 1D). The joint tissues from vehicle-treated mice exhibited infiltrated lymphocytes, but butyrate treatment restored the level of fat in the joint. CD4^+^ T cell infiltration in synovial tissues was markedly reduced by butyrate treatment. Finally, butyrate markedly reduced the expression of inflammatory cytokines by various cell types, demonstrating its suppressive effect on CIA.

### Butyrate Inhibits Osteoclastogenesis

**Figure 2:**
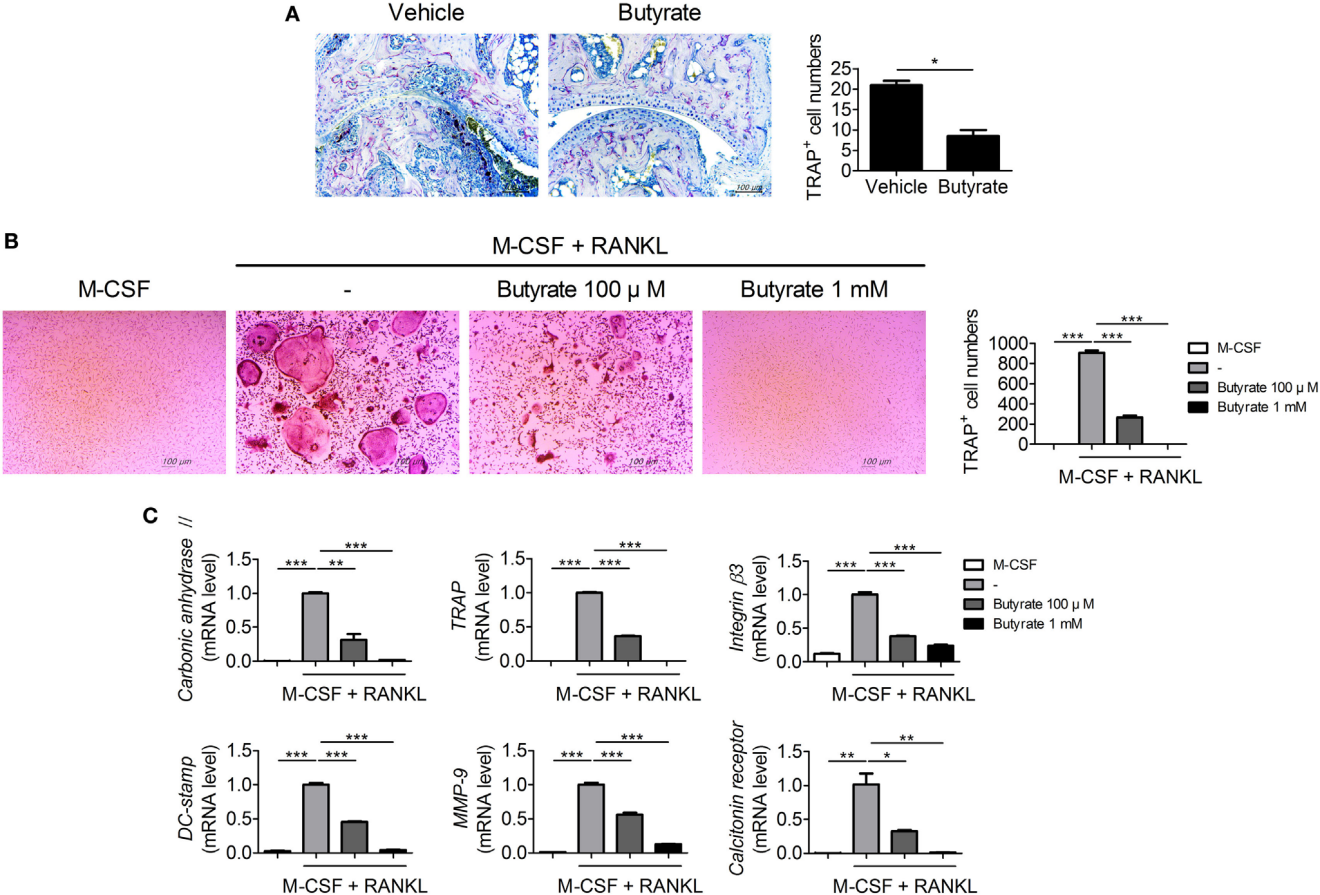
Butyrate inhibits osteoclast formation. **(A)** Synovial tissue sections were stained with anti- tartrate-resistant acid phosphatase (TRAP) antibodies (original magnification, 200×); numbers of TRAP-stained cells are shown (*n* = 3). **(B)** Collagen-induced arthritis mouse bone marrow cells were isolated and cultured with 10 ng/mL M-CSF and/or 50 ng/mL RANKL in the presence or absence of 100 µM and 1 mM butyrate to induce differentiation into osteoclasts, which were fixed and stained for TRAP. Representative photographs are shown (original magnification, 100×). **(C)** The messenger RNA levels of carbonic anhydrase II, TRAP, integrin β3, dendritic cell-specific transmembrane protein (DC-stamp), matrix metalloproteinase-9, and calcitonin receptor were determined by real-time polymerase chain reaction. *p < 0.05, **p < 0.01, ***p < 0.001.

Bone destruction, which is mediated by osteoclasts, is directly related to the prognosis of RA patients. Therefore, we assessed the effect of butyrate on osteoclastogenesis. Inhibition of HDAC suppresses osteoclastogenesis by enhancing IFN-β production (18). *In vivo* treatment with butyrate markedly reduced expression of TRAP, a marker of osteoclasts, in synovial tissue from CIA mice (Figure 2A). Mouse bone-marrow cells were stimulated with M-CSF and RANKL to induce osteoclastogenesis with or without butyrate; butyrate inhibited the formation of osteoclasts *in vitro* (Figure 2B). The mRNA levels of the osteoclastogenic markers including carbonic anhydrase II, TRAP, integrin β3, DC-stamp, MMP-9, and calcitonin receptor were significantly decreased by butyrate treatment in a dose-dependent manner (Figure 2C). In particular, 1 mM butyrate treatment suppressed the expression of these markers almost completely.

### Butyrate Controls Glucocorticoid Receptors by Inhibiting HDAC2, Resulting in Induction of SLPI and Attenuation of Osteoclastogenesis

**Figure 3:**
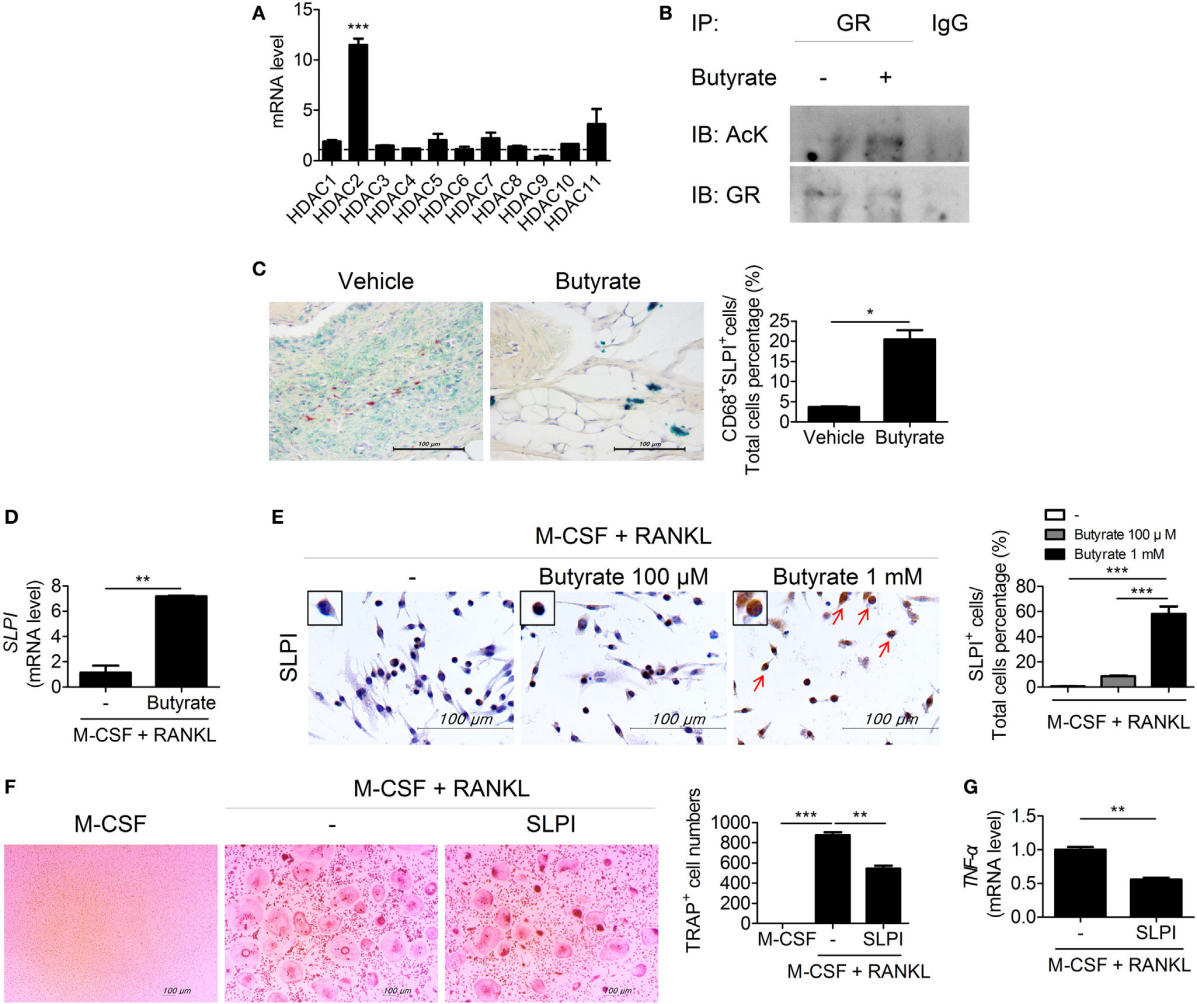
Butyrate inhibits osteoclastogenesis by inhibiting HDAC2. **(A)** Bone marrow cells were isolated from collagen-induced arthritis (CIA)-induced mice and differentiated into osteoclasts in the presence of M-CSF and RANKL. After 4 days, the messenger RNA (mRNA) level of each subtype of histone deacetylases was measured. Dotted line indicates the expression of M-CSF single stimulation condition. **(B)** RAW 264.7 cells were incubated with butyrate under OC differentiation conditions for 1 day, immunoprecipitated with an anti-glucocorticoid receptor (GR) antibody, and immunoblotted using anti-acetyl lysine and anti-GR antibodies. **(C)** Representative histological features of the joints of saline-treated CIA mice and butyrate-treated mice (*n* = 3). Immunohistochemical staining for CD68 and secretory leukocyte protease inhibitor (SLPI) is shown. The percentage of CD68 and SLPI co-stained cells among total cells is shown. CD68, red; SLPI, green; overlap, blue, or purple (original magnification, 400×). **(D)** Relative SLPI mRNA levels in butyrate-treated samples when osteoclasts were differentiated into BMs in CIA mice. **(E)** Immunohistochemical staining for SLPI in osteoclasts differentiated from bone-marrow cells of CIA mice. Dyed cells are colored brown (original magnification, 400×). Graph shows the numbers of cells expressing SLPI. ***p < 0.001 (right panel). **(F)** CIA mouse bone-marrow cells were isolated and cultured with 10 ng/mL M-CSF and/or 50 ng/mL RANKL in the presence or absence of 100 ng/mL SLPI to induce differentiation into osteoclasts, which were fixed and stained for tartrate-resistant acid phosphatase. Representative photographs are shown (original magnification, 100×). **(G)** TNF-α mRNA level as determined by real-time polymerase chain reaction. *p < 0.05, **p < 0.01, ***p < 0.001.

Histone deacetylases inhibitors suppress osteoclastogenesis and reduce bone destruction (18, 19) and butyrate inhibits HDACs. We thus investigated the mRNA level of each HDAC subtype in osteoclastogenesis-induced bone-marrow cells from CIA mice. The mRNA level of *HDCA2* was increased during osteoclastogenesis (Figure 3A). Deacetylation of GR by HDAC2 results in repression of GR-mediated gene expression (20). Interestingly, acetylation-dependent GR increases the transcription of SLPI, which suppresses inflammation and joint damage in arthritis by downregulating TNF-α (21). Thus, we evaluated the effect of butyrate on the expression of SLPI *via* GR. Acetylation of GR in RAW 264.7 mouse macrophages cultured under osteoclast differentiation conditions was increased by butyrate treatment (Figure 3B). In the joint tissues of CIA-induced mice, the percentage of cells co-staining for CD68 and SLPI was significantly higher in the butyrate-treated group compared to the vehicle group (Figure 3C). *In vitro* treatment with butyrate increased the SLPI mRNA (Figure 3D) and protein (Figure 3E) levels in osteoclasts differentiated from BMMs of CIA mice, and the addition of recombinant SLPI inhibited osteoclastogenesis (Figure 3F) and decreased the TNF-α mRNA level (Figure 3G). TNF-α directly induces osteoclastogenesis (22, 23); therefore, the butyrate-mediated increase in SLPI expression and decrease in TNF-α expression could lead to inhibition of osteoclastogenesis. Butyrate suppresses osteoclastogenesis by inhibiting HDAC2, which results in upregulation of the activity of GR and its substrate SLPI.

### Butyrate Controls the T_H_17/T_reg_ Balance

**Figure 4:**
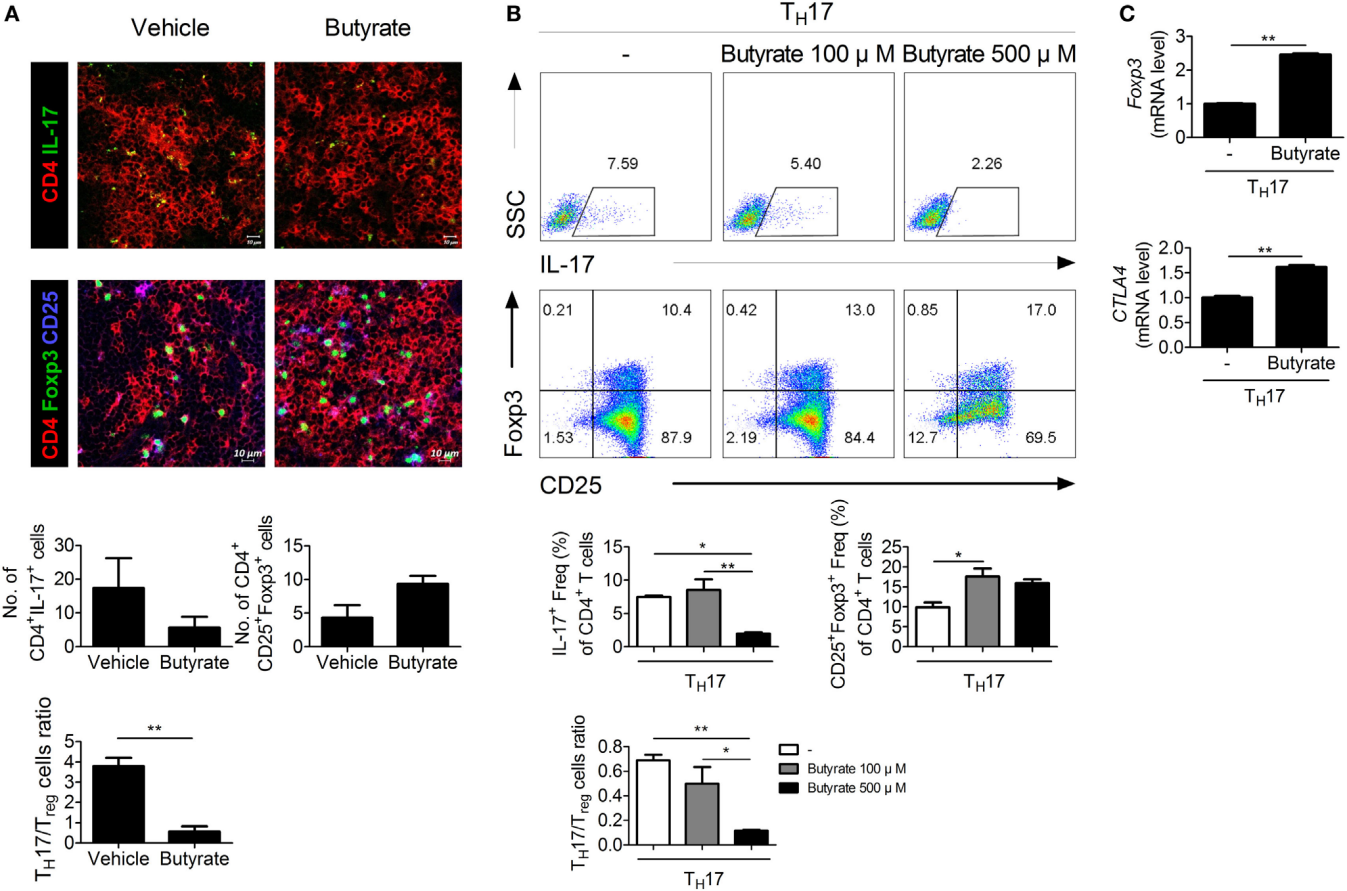
Butyrate regulates T_H_17/T_reg_ cells in mouse T cells. **(A)** Confocal microscopy of spleen cryosections, stained for CD4 and IL-17 (T_H_17 cells) or CD4, CD25, and Foxp3 (T_reg_ cells) (original magnification, 400×). The numbers and ratios of positive cells are shown (*n* = 3). **p < 0.01. **(B)** CD4^+^CD62L^high^CD44^low^-naïve CD4^+^ T cells were extracted from the spleens of C57BL/6 mice, cultured with butyrate under T_H_17-polarizing conditions for 72 h, and subjected to flow cytometry analysis of IL-17- or Foxp3-expressing cells. The representative results from three independent experiments are shown in the upper panel. Relative bar charts are shown in the lower panel. *p < 0.05, **p < 0.01. **(C)** Two days after inducing T_H_17 cells, the messenger RNA levels of T_reg_ markers were measured. *p < 0.05, **p < 0.01.

The imbalance between T_H_17 and T_reg_ cells is important in RA (24–26). To assess the effect of *in vivo* treatment with butyrate on the population of T_H_17 or T_reg_ cells, these cells were identified in the spleens of butyrate- or vehicle-treated CIA mice using confocal microscopy (Figure 4A). The number of IL-17-expressing CD4^+^ T cells, which are considered T_H_17 cells, was significantly reduced by butyrate treatment, but the number of Treg (CD4^+^CD25^+^Foxp3^+^) cells was increased (Figure 4A). As a result, the T_H_17/Treg ratio was decreased. The expression of IL-17 and Foxp3 by naïve CD4^+^ T cells cultured under T_H_17 differentiation conditions was reduced and increased, respectively, by *in vitro* treatment with butyrate (Figure 4B). Butyric acid did not show any cellular toxicity (Figure S2 in Supplementary Material). The T_H_17/T_reg_ ratio was decreased by butyrate treatment in a dose-dependent manner (Figure 4B). The increased number of T_reg_ cells was confirmed by the increased mRNA levels of *CTLA4* and *Foxp3* (Figure 4C). Interestingly, the level of Tyr705- or Ser727-phosphorylated STAT3, another T_H_17/T_reg_ controller, was not changed (Figure S1 in Supplementary Material). Therefore, butyrate regulates the T-cell subtype balance irrespective of STAT3 phosphorylation; thus, it may have potential as a therapeutic agent for RA.

### Butyrate Inhibits HDAC8 During T_H_17 Development, Induces CPTI, and Suppresses NR1D1 by Transactivating Estrogen-Related Receptor Alpha

**Figure 5:**
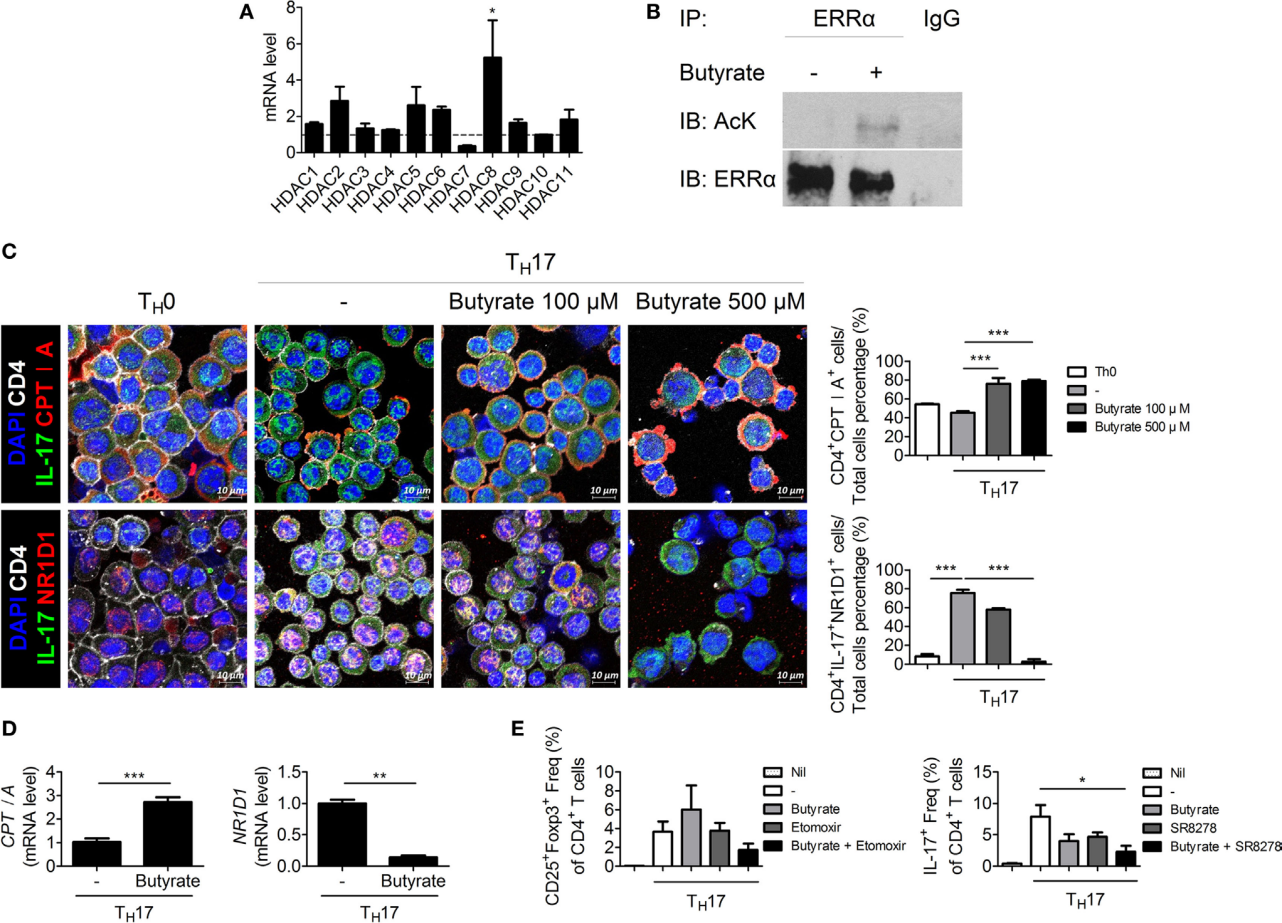
Butyrate regulates T cells by inhibiting HDAC8. **(A)** Naïve CD4^+^ T cells were cultured under T_H_17-polarizing conditions for 1 day, and the messenger RNA (mRNA) level of each subtype of histone deacetylases was measured. Dotted line indicates the expression of conditions that did not stimulate anything. **(B)** EL4 cells were stimulated with butyrate, anti-CD3, and IL-6; immunoprecipitated with an anti-ERRα antibody; and immunoblotted using anti-acetyl lysine and anti-ERRα antibodies. **(C)** Naïve CD4^+^ T cells were cultured under T_H_17-polarizing conditions with 500 µM butyrate for 3 days, and CPTIA and NR1D1 expression was visualized by confocal microscopy. Relative bar charts are shown in the right panel. ***p < 0.001. **(D)** One day after induction of T_H_17 cells, the mRNA levels of *CPTIA* and *NR1D1* were measured. **(E)** Naïve CD4^+^ T cells were isolated from splenocytes and cultured with butyrate, etomoxir, or SR8278 under T_H_17 differentiation conditions for 3 days, and the T_H_17 and T_reg_ cell populations were analyzed by flow cytometry. *p < 0.05.

To elucidate the mechanism by which butyrate affects T_H_17 cells, we next attempted to identify a T_H_17 cell-specific HDAC subtype. HDAC8 was highly expressed during T_H_17 cell differentiation (Figure 5A). HDAC8 deacetylates estrogen-related receptor alpha (ERRα) and enhances its transcriptional activity (27). ERRα is induced upon T-cell activation and controls the growth and proliferation of effector T cells (28). Butyrate treatment increased the acetylated ERRα level in EL4 mouse lymphocytes cultured under T_H_17 differentiation conditions (Figure 5B). The decreased transcriptional activity of ERRα leads to an increase in the expression of carnitine palmitoyltransferase I (CPTI) (28) and a decrease in that of nuclear receptor subfamily 1, group D, member 1 (NR1D1, also known as Rev-ErbA alpha) (29). Treatment of CD4^+^ T cells with etomoxir, a specific inhibitor of CPTI, reduces the T_reg_ population (30). Naïve NR1D1*^−/−^* CD4^+^ T cells have a decreased ability to differentiate into T_H_17 cells compared to WT T-cells (31). Likewise, CPTI and NR1D1 play an important role in T_reg_ and T_H_17 cells, respectively. We focused on CPTIA (also known as CPTI-L, liver isoform), which is expressed in all cells except skeletal muscle cells and brown adipose cells (32, 33). After butyrate treatment, confocal microscopy revealed that the expression of CPTIA was increased and that of NR1D1 was decreased in T_H_17 cells differentiated from naïve CD4^+^T cells (Figure 5C). The CPTIA and NR1D1 mRNA levels were also increased and decreased, respectively (Figure 5D). Next, naïve CD4^+^T cells were cultured under T_H_17 differentiation conditions and treated with butyrate in the presence of etomoxir and SR8278 [inhibitors of CPTI and NR1D1, respectively (34)]. The number of T_reg_ and T_H_17 cells was increased and decreased by butyrate, respectively, and these effects were enhanced in the presence of the two inhibitors (Figure 5E). Therefore, butyrate regulates the T_H_17/T_reg_ cell balance by inhibiting HDAC8 through its effects on ERRα.

### Therapeutic Effect of Butyrate Is Mediated by IL-10

**Figure 6:**
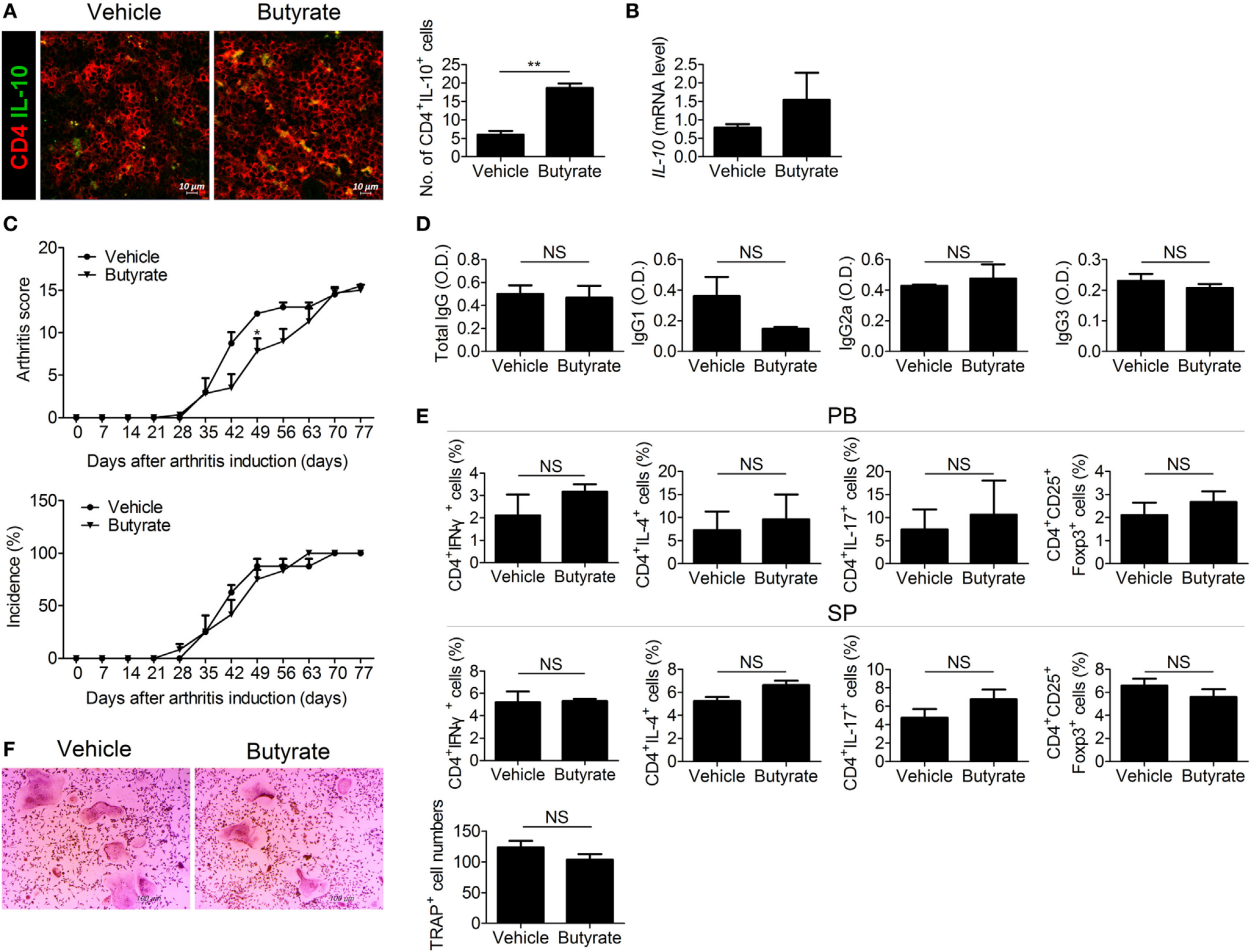
The therapeutic effect of butyrate is mediated *via* IL-10. **(A)** Confocal microscopy of cryosections of the spleens of collagen-induced arthritis (CIA) mice stained for CD4 and IL-10 (original magnification, 400×). Numbers of positive cells are shown (*n* = 3). **(B)** IL-10 messenger RNA level in the spleens of CIA-induced DBA/1J mice as determined by real-time polymerase chain reaction. **(C)** CII-injected IL-10-KO mice were administered 100 mg/kg butyrate intraperitoneally three times per week 3 days after secondary immunization (*n* = 3). The clinical score (upper panel) and incidence (lower panel) of arthritis are shown. **(D)** Serum concentrations of the CII-specific total IgG, IgG1, IgG2a, and IgG3 levels of CIA-induced IL-10-KO mice. **(E)** Flow cytometry analysis of the T_H_1, T_H_2, T_H_17, and T_reg_ cell populations in peripheral blood (upper panel) or splenocytes (lower panel) from CIA-induced IL-10-KO mice. **(F)** Bone marrow cells of CII-injected IL-10-KO mice were isolated and differentiated into osteoclasts (*n* = 3). After 5 days, the cells were stained for tartrate-resistant acid phosphatase (TRAP) expression; representative photographs are shown (original magnification, 100×). The number of multinucleated TRAP-positive cells was also determined. *p < 0.05, **p < 0.01.

Butyrate exerts an anti-inflammatory effect by downregulating IL-12 and upregulating IL-10 (12). Some butyrate*-*producing *Clostridium* species are involved in T_reg_ development. IL-10-producing T_reg_ cells increase in number in mice colonized by a mixture of *Clostridiales* (35). Therefore, we assessed the role of IL-10 in the therapeutic effect of butyrate. The number of IL-10-expressing cells (Figure 6A) and the IL-10 mRNA level (Figure 6B) were increased in the spleens of CIA mice treated with butyrate. However, butyrate treatment did not exert a significant therapeutic effect in CIA-induced IL-10-KO mice (Figure 6C). Butyrate also had little effect on the serum levels of CII-specific IgGs (Figure 6D). In the absence of IL-10, butyrate did not significantly alter the effector CD4^+^ T-cell subtype populations in the peripheral blood or spleen (Figure 6E). IL-10 inhibits bone destruction by reducing NFATc1 expression (36, 37). Therefore, we also investigated the effect of butyrate on osteoclastogenesis using BMMs from IL-10-KO mice; in the absence of IL-10, butyrate did not suppress osteoclastogenesis and did not exert a significant effect on the number of TRAP-positive MNCs (Figure 6F). Taken together, our data demonstrate that the therapeutic activity of butyrate in CIA mice is dependent on IL-10.

### Effect of Butyrate on Human T_H_17/T_reg_ Balance and Osteoclastogenesis

**Figure 7:**
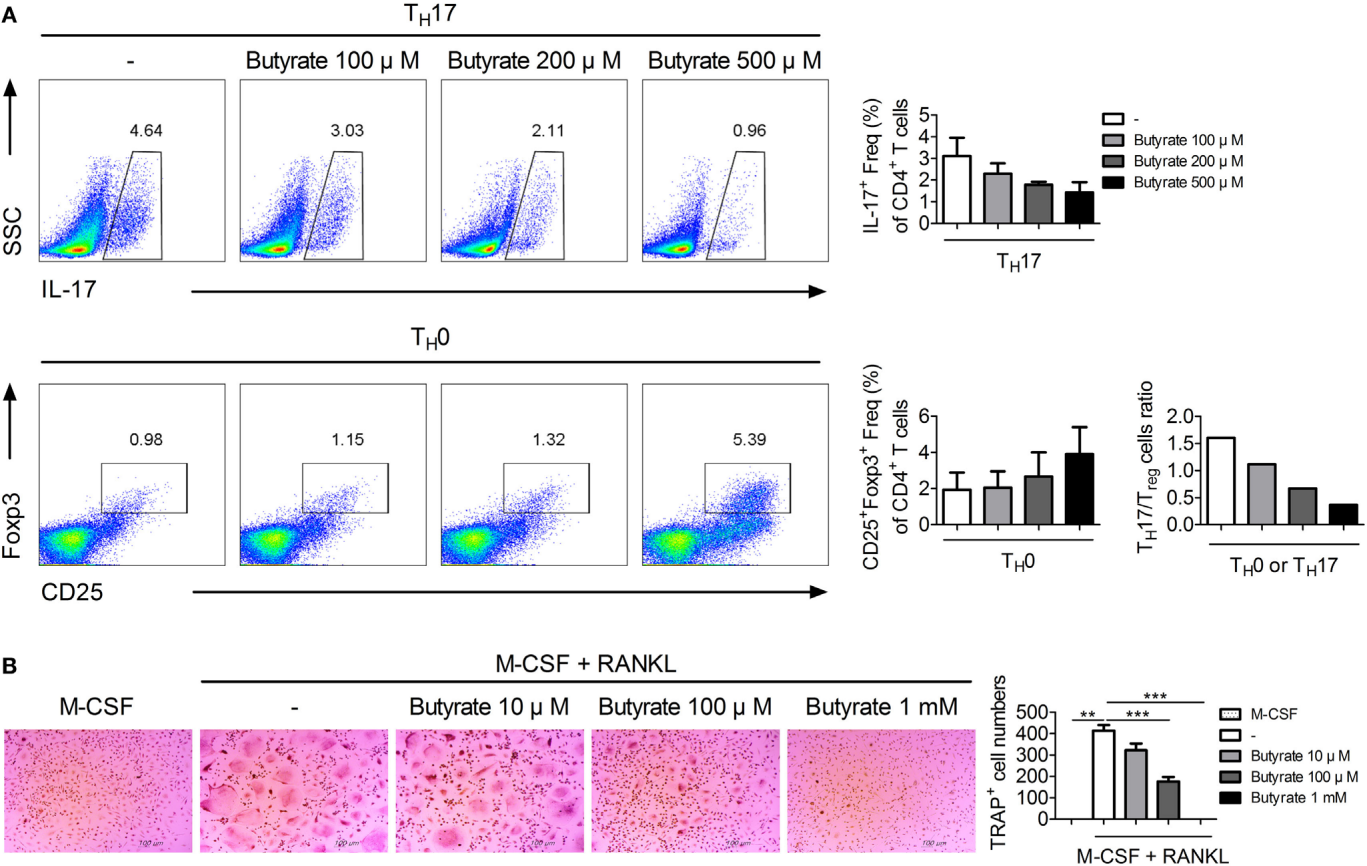
Butyrate has potential as a therapeutic agent for rheumatoid arthritis. **(A)** CD4^+^ T cells were isolated from normal human peripheral blood mononuclear cells (PBMCs) and cultured with butyrate under T_H_17- or T_H_0-polarizing conditions for 72 h. T_H_17 cells (CD4^+^IL-17^+^) and T_reg_ cells (CD4^+^CD25^+^Foxp3^+^) were subjected to flow cytometry analysis. Numbers and ratios of IL-17- or Foxp3-positive cells are shown. **(B)** Human PBMCs were differentiated into osteoclasts, fixed, and stained for tartrate-resistant acid phosphatase; representative photographs are shown (original magnification, 100×). **p < 0.01, ***p < 0.001.

We investigated the effect of butyrate on the human T_H_17/T_reg_ balance and osteoclastogenesis *in vitro*. Normal human CD4^+^ T cells were isolated and cultured under T_H_17 cell-polarizing conditions with or without butyrate, and the T-cell subtype populations were analyzed by flow cytometry. The numbers of T_H_17 and T_reg_ cells were dose-dependently reduced and increased, respectively, by butyrate. Also, the T_H_17/T_reg_ ratio was decreased by butyrate in a dose-dependent manner, suggesting the regulation of the T_H_17/T_reg_ balance by butyrate (Figure 7A). In agreement with the results using mouse cells, treatment of human PBMCs with butyrate inhibited M-CSF and RANKL-induced osteoclastogenesis (Figure 7B). Therefore, butyrate shows potential for the treatment of RA.

## Discussion

Disturbed gene expression caused by epigenetic modification can directly or indirectly lead to the development of disease. Acetylation and deacetylation are epigenetic modifications that regulate gene transcription and protein functions and are mediated by histone acetyltransferases and HDAC. For this reason, inhibitors of HDAC are used clinically to treat various conditions (38, 39). Epigenetic modification, including acetylation, of cellular proteins is involved in the pathogenesis of RA (40); this operates by modulating the activity of signaling pathways or the transcription of key factors, such as T-bet, Gata3, RORγt, and Foxp3, which influence the fate of effector T cells. In other words, the orchestration of gene expression or protein modification in effector T cells may be associated with autoimmune diseases. Like butyrate, the pan-HDAC inhibitors SAHA and TSA exert therapeutic effects in RA (41, 42), but their targets and mechanisms are unclear.

Abnormal osteoclast differentiation is a major cause of RA. The gut microbiota influences bone metabolism through various pathways (5). T_H_17 cells are involved in the development of various autoimmune diseases, including RA, and maintaining the T_H_17 and T_reg_ balance is a challenge in the treatment of autoimmune diseases (43, 44). Microbiota is now considered important factor in T-cell homeostasis and is implicated in inflammatory diseases (45). Butyrate ameliorates autoimmune diseases (e.g., experimental allergic encephalomyelitis, GvHD, and ulcerative colitis) and is the subject of clinical trials (13, 46–49). Also, in animal models, butyrate inhibits inflammation induced by butyrate-producing Clostridia, such as *F. prausnitzii* and *Butyricicoccus pullicaecorum* (50, 51). However, few studies have focused on the therapeutic effect of butyrate on RA. Here, we report that butyrate exerts a therapeutic effect in an animal model of RA and recovers the T_H_17/T_reg_ imbalance and osteoclastogenesis. We also assessed the mechanism(s) underlying the anti-arthritic effect of butyrate.

In CIA mice, parameters related to joint destruction, inflammation, expression of proinflammatory cytokines, and osteoclast formation were recovered by butyrate treatment (Figures 1 and 2). Moreover, we elucidated the mechanism by which butyrate modulates osteoclastogenesis and effector T-cell differentiation, which are critical in the pathogenesis of RA.

Deletion of HDAC7 and HDAC3 decreases and increases, respectively, bone destruction (52, 53). HDAC9 inhibits osteoclastogenesis *via* a different pathway than HDAC3 (54). In this study, the expression of HDAC2 was increased during osteoclastogenesis (Figure 3). HDAC2 upregulates RANKL-induced osteoclastogenesis by activating Akt, which results in FoxO1 depletion and interference of HDAC2 abrogates TRAP-positive osteoclasts *in vitro* (55). Butyrate reduced TRAP expression and proposed pathway of HDAC2-related GR-SLPI axis affecting osteoclastogenesis. Akt negatively regulates GR expression (56); thus, inhibition of the HDAC2-related pathway may interrupt the Akt-related pathway, resulting in osteoclast differentiation. Butyrate inhibited expression of HDAC2, which inhibits transcription of GR by deacetylation, leading to upregulation of SLPI expression. SLPI protects epithelial tissue from serine proteases (57–60), and reportedly reduces inflammation and joint damage in arthritis (21). This is the first report that inhibition of HDAC2 by butyrate upregulates SLPI expression in osteoclasts.

HDAC8 is expressed specifically during T_H_17 development, and inhibition of HDAC8 induces apoptosis of T-cell lymphoma cells (61). Its target ERRα is related to immune responses involving effector T cells and macrophages. ERRα controls the development of effector T cells, and suppression of its expression in T cells ameliorates experimental autoimmune encephalomyelitis (28). ERRα negatively regulates toll-like receptor-induced inflammation, and ERRα-deficient mice are susceptible to endotoxin-induced septic shock (62). Therefore, reduced deacetylation of ERRα by butyrate may ameliorate autoimmune diseases. In our study, butyrate decreased and increased the numbers of T_H_17 and T_reg_ cells, respectively, and exerted a therapeutic effect in a mouse model of arthritis by modulating the T_H_17/T_reg_ balance *in vivo* and *in vitro* (Figure 4). Regulation of the T_H_17/T_reg_ balance by butyrate did not involve phosphorylation of STAT3, a regulator of the T_H_17/T_reg_ balance (Figure S1 in Supplementary Material); thus, its effect was mediated by some other pathway. Inhibition of HDAC8 by butyrate induced ERRα acetylation, which reduced transcription of ERRα, leading to an increase in the expression of CPTI and a decrease in that of NR1D1. CPTI and NR1D1 were involved in the regulation of T-cell populations by butyrate (Figure 5). Control of the T_H_17/T_reg_ balance by butyrate may have influenced the expression level of IL-10, leading butyrate to lose its therapeutic effect in IL-10 KO mice (Figure 6). Additionally, butyrate modulated the T-cell populations in human PBMCs and inhibited osteoclastogenesis (Figure 7), thus showing potential for treatment of RA.

**Figure 8:**
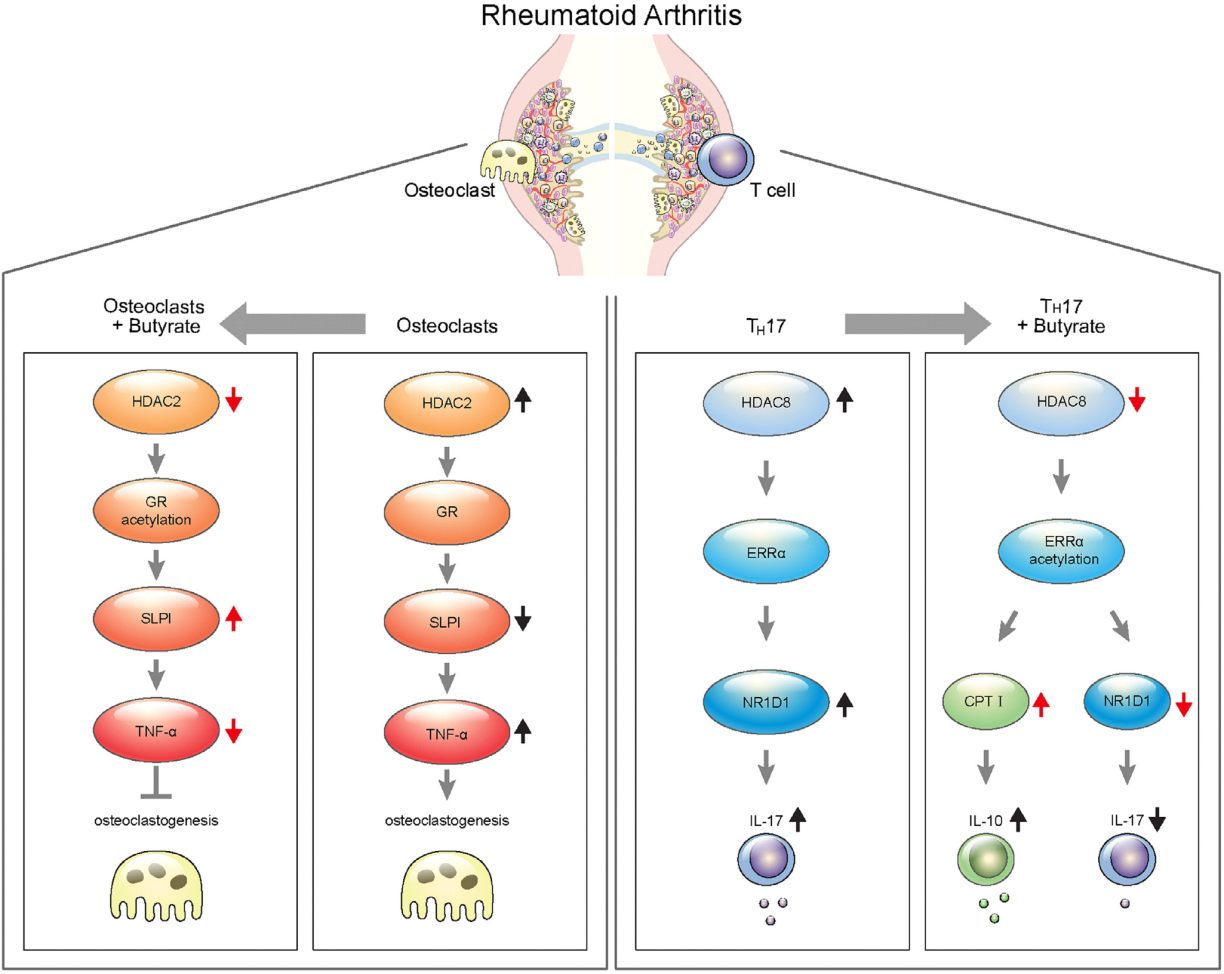
Schematic illustration of targeting for osteoclastogenesis and T_H_17 cells by butyrate in rheumatoid arthritis.

Collectively, these findings suggest that sodium butyrate, a metabolite of the gut microbiota, ameliorates rheumatoid inflammation by targeting HDAC2 in osteoclasts and HDAC8 in T cells. Therefore, sodium butyrate shows promise for the treatment of RA (Figure 8).

## Ethics Statement

The Animal Care Committee of The Catholic University of Korea approved the experimental protocol. All experimental procedures were evaluated and carried out in accordance with the protocols approved by the Animal Research Ethics Committee at the Catholic University of Korea (approval numbers, 2017-0070-02 and 2018-0014-01). All procedures performed followed the ethical guidelines on animal use. Approval by the ethics committee of Seoul St. Mary’s Hospital (Seoul, Republic of Korea) was obtained for all procedures. All human experimental procedures were approved by the Ethics Committee of Seoul St. Mary’s Hospital (Seoul, Republic of Korea, KC17TNSI0570).

## Author Contributions

DK, J-EK, EK, M-LC, and S-KK designed the experiments and analyzed the data. DK performed *in vitro* assays with help from EK and M-JP. DK and J-GR performed the animal experiments. EK, K-AJ, and J-WC conducted immunohistochemistry experiments. DK and J-EK wrote the manuscript along with input from SL, EK, Y-MM, M-LC, and S-KK. DK, J-EK, EK, M-JP, S-HP, M-LC, and S-KK discussed and developed the study concept. All authors critically reviewed and approved the final form of the manuscript.

## Conflict of Interest Statement

Author K-AJ was employed by company Impact Biotech. All other authors declare no competing interests.

## Supplementary Material

Figure S1 Regulation of T cells by butyrate is independent of phosphorylation of STAT3. **(A)** pSTAT3 Y705, pSTAT3 S727, and STAT3 expression in splenocytes from C57BL/6 mice was analyzed by western blotting; a representative figure is shown. **(B)** pSTAT3 Y705, pSTAT3 S727, and STAT3 expression in CD4^+^ T cells from C57BL/6 mice was analyzed by western blotting; a representative figure is shown.

Figure S2 The viability of naïve T cells from normal mice treated with butyrate. The cell viability was measured using CCK assay after treatment with indicated concentrations of butyrate.
