# Supplementary figures and images for: Attenuation of Rheumatoid Inflammation by Sodium Butyrate Through Reciprocal Targeting of HDAC2 in Osteoclasts and HDAC8 in T Cells
Source: Front Immunol. 2018 Jul 6;9:1525. doi: 10.3389/fimmu.2018.01525 (PMC6043689; doi:10.3389/fimmu.2018.01525)

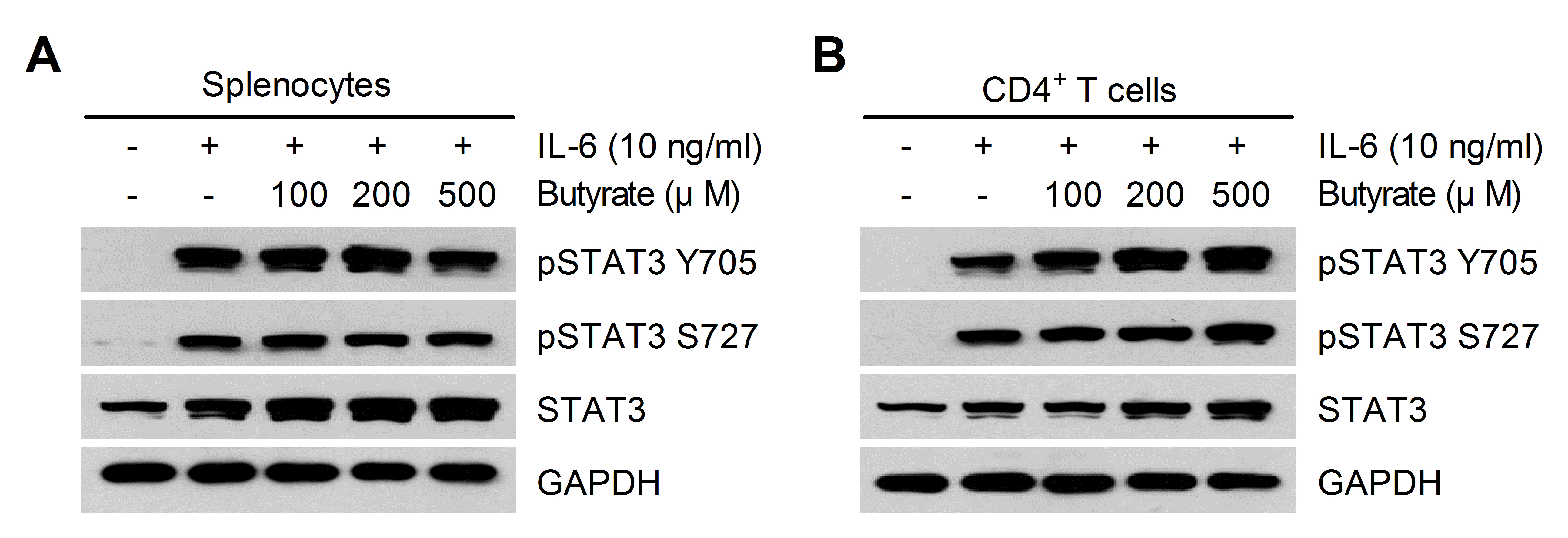

Supplement: Figure S1 — Regulation of T cells by butyrate is independent of phosphorylation of STAT3. (A) pSTAT3 Y705, pSTAT3 S727, and STAT3 expression in splenocytes from C57BL/6 mice was analyzed by western blotting; a representative figure is shown. (B) pSTAT3 Y705, pSTAT3 S727, and STAT3 expression in CD4+ T cells from C57BL/6 mice was analyzed by western blotting; a representative figure is shown. [file image_1.jpeg]

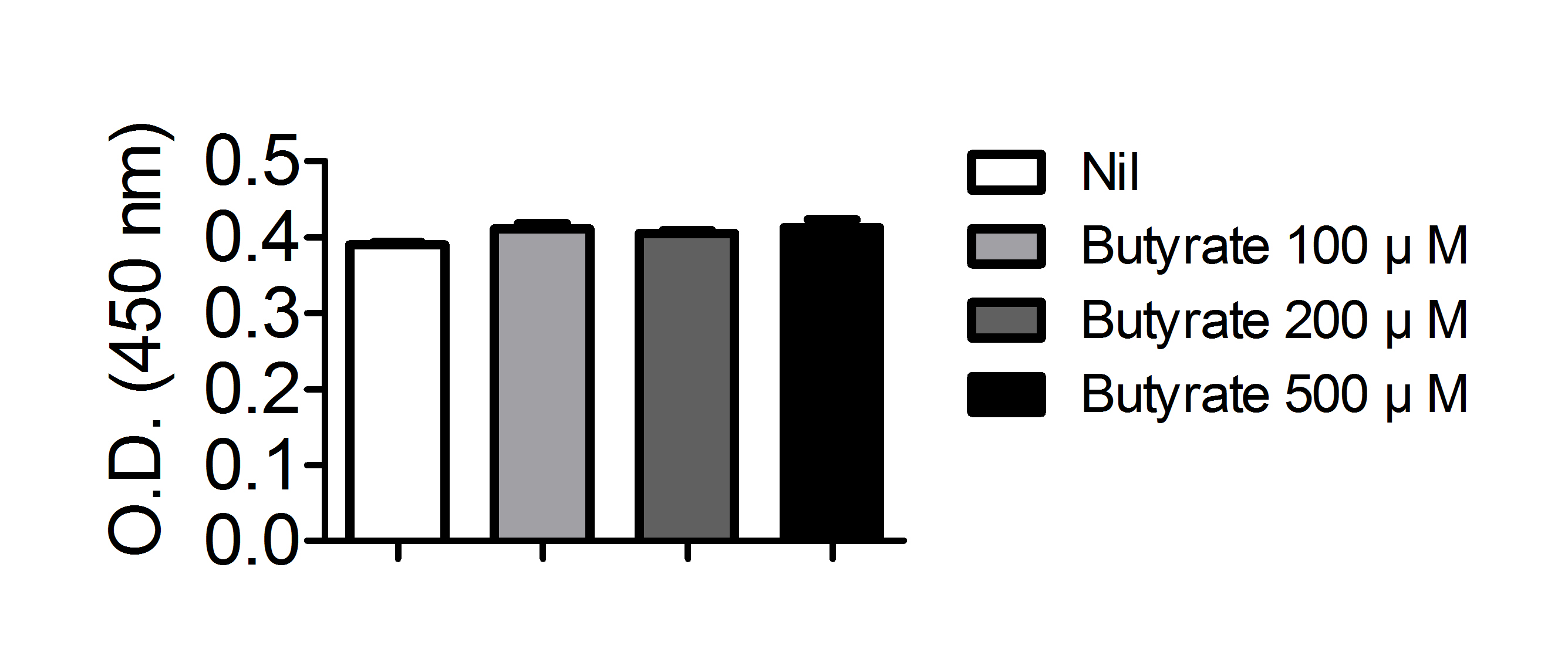

Supplement: Figure S2 — The viability of naïve T cells from normal mice treated with butyrate. The cell viability was measured using CCK assay after treatment with indicated concentrations of butyrate. [file image_2.jpeg]
